# Premedication with oral paracetamol for reduction of propofol injection pain: a randomized placebo-controlled trial

**DOI:** 10.1186/s12871-019-0758-y

**Published:** 2019-06-11

**Authors:** Sasikaan Nimmaanrat, Manasanun Jongjidpranitarn, Sumidtra Prathep, Maliwan Oofuvong

**Affiliations:** Department of Anesthesiology, Faculty of Medicine, Prince of Songkla University, Hatyai, Songkhla 90110 Thailand

**Keywords:** Paracetamol, Propofol, Pain, Injection pain

## Abstract

**Background:**

To compare the effect of premedication with 2 different doses of oral paracetamol to prevent pain at propofol intravenous injection.

**Methods:**

We conducted a double-blind randomized controlled trial in which patients scheduled for induction of general anesthesia with intravenous propofol received either a placebo, 500 mg or 1000 mg of oral paracetamol (P500 and P1000, respectively) 1 h prior to induction. Two mg/kg of propofol was injected at a rate of 600 ml/hr. After 1/4 of the full dose had been injected, the syringe pump was paused, and patients were asked to rate pain at the injection site using a verbal numerical rating score (VNRS) from 0 to 10.

**Results:**

Three hundred and twenty-four patients were included. Pain intensity was lower in both P500 and P1000 groups (median VNRS [interquartile range] = 2 [0–3] and 4 [2–5], respectively) than in the placebo group (8 [7–10]; *P* < 0.001)*. The rate of pain was lower in the P1000 group (70.4%) than in both the P500 and the placebo group (86.1 and 99.1%, respectively; *P* < 0.001)*. The respective rates of mild (VNRS 1–3), moderate (VNRS 4–6) and severe pain (VNRS 7–10) were 47.2, 23.2 and 0% in the P1000 group, 28.7, 50 and 7.4% in the P500 group, and 0, 22.2 and 76.9% in the placebo group (*P* < 0.001* for between group comparisons). Tolerance was similar in the 3 groups.

**Conclusions:**

A premedication with oral paracetamol can dose-dependently reduce pain at propofol intravenous injection. To avoid this common uncomfortable concern for the patients, this well-tolerated, available and cheap treatment appears as an option to be implemented in the current practice.

**Trial registration:**

TCTR20150224002. Prospectively registered on 24 February 2015**.**

## Background

Propofol (di-isopropylphenol) is the most frequently used agent for the induction of general anesthesia because of its rapid onset and short duration of action. However, pain from the injection is a common problem [1]. The incidence of injection pain has been shown to vary between 28 and 90% which might be severe [2, 3] and the data from Songklanagarind Hospital found the high incidence of pain as 83%.

Pain upon injection of some anesthetic agents are thought to be a direct irritant effect by the non-physiological osmolality or pH of their preparations [4]. Nonetheless, propofol is nearly isotonic, nonhyperosmolar and has a pH from 6 to 8.5. Hence, this concept cannot explain for the pain produced by the injection of propofol [1]. Propofol injection pain may be caused by an effect via the kinin cascade [5]. In addition, many factors seem to contribute to the incidence of injection pain including site [6] and speed of injection [7], size of vein [7, 8], rate of intravenous fluid infusion [9], concentration of propofol in the aqueous phase [4] as well as blood buffering effects [10].

A number of approaches have been proposed to lessen the injection pain such as injection of propofol at an antecubital fossa, fast injection [7] and pretreatment with lidocaine [11], opioids [12], or non-steroidal anti-inflammatory drugs (NSAIDs) [13]. The effective technique is a combination of lidocaine pretreatment together with venous occlusion (a modified Bier’s block) [3]. However, this inflated arm tourniquet technique is quite difficult. From a systematic review and meta-analysis, the most 2 effective procedures to decrease propofol injection pain are injecting through an antecubital vein and pretreatment with lidocaine together with venous occlusion when a hand vein is used [14].

Canbay et al. [15] showed that intravenous acetaminophen (paracetamol) could diminish injection pain. The incidence of pain was significantly reduced to 22% as compared to a control group but less than lidocaine. Borazan et al. [16] compared the effect of injection of different paracetamol doses with lidocaine. They found that paracetamol 2 mg/kg administered intravenously 1 min before propofol was more effective than paracetamol 1 mg/kg and lidocaine in reducing propofol injection pain. The issue of pain at propofol injection pain should be addressed and managed accordingly. We hypothesized that oral paracetamol can reduce the severity of propofol injection pain. Our primary endpoint was pain intensity measured by verbal numerical rating score upon propofol injection. We used oral form of paracetamol because it is easier to administer and much cheaper in comparison to intravenous injection.

Additionally, Seymour et al. [17] demonstrated that a 1000-mg dose was more effective than 500 mg in reducing postoperative pain after third molar surgery. In regard to this study, we aimed to compare the efficacy of paracetamol 500 mg versus 1000 mg for reduction of propofol injection pain.

## Methods

This study was a double-blinded randomized controlled trial (RCT). It was approved by the Faculty of Medicine, Prince of Songkla University Ethics Committee and registered with Thai Clinical Trial Registry (TCTR20150224002: prospectively registered on February 24, 2015). The principal investigator was Dr. Nimmaanrat. The data were collected from June 2015 until February 2016 at Songklanagarind Hospital (Faculty of Medicine, Prince of Songkla University). The authors prepared this trial report in accordance to the Consolidated Standards of Reporting Trials (CONSORT) guidelines. The full protocol is accessible on request.

We recruited 324 patients with the American Society Anesthesiologists (ASA) physical status I-III of who were aged between 18 and 65, scheduled for elective surgeries under general anesthesia, and having an intravenous catheter number 20G at a hand dorsum.

Exclusion criteria included weight less than 50 kg, chronic pain, hypertension, cardiovascular disease or cerebrovascular disease, difficulty in communicating, cirrhosis or abnormal liver function test result (aspartate transaminese (AST), alanine transaminase (ALT) ≥ 2 times of normal range), renal failure or creatinine clearance (CrCl) ≤ 10 umol/L, paracetamol and/or propofol allergy. Exclusion criteria also included patients who were not using propofol for an induction, using an intravenous catheter that was not on a hand dorsum, or whereas the size of the catheter was not 20G and had to have a rapid sequence induction.

After obtaining a written informed consent, a randomization was performed by using a block of 6 method. The drugs were prepared by one of the investigators (MJ), with both the patient and an independent assessor (anesthesiologist in-charge) blinded. The groups Pb, P500 and P1000, patients were premedicated with oral placebo, 500 or 1000 mg of paracetamol, respectively 1 h prior to transferal to the operating room. Each patient received either 2 tablets of placeco (Pb group), 1 tablet of placebo and 1 tablet of paracetamol 500 mg (P500), or 2 tablets of paracetamol 500 mg (P1000). Both placebo and paracetamol were identical in shape, size, color and weight. None of them received any other analgesic or sedative drug. A 20G intravenous catheter was inserted into a superficial vein on the hand dorsum and intravenous fluid at a rate of 80 ml/hr. was infused into each patient.

After preoxygenation, an emulsion of 1% propofol in a mixture of long-chain and medium-chain triglycerides (Lipuro®, B Braun) 2 mg/kg (for obese patients, dose was calculated by using lean body weight) was intravenously administered into each patient with a syringe pump at a rate of 600 ml/hr. (10 ml/min). After 1/4 of the calculated dose of propofol had been delivered, the infusion pump was temporarily paused and the patient was asked to rate his/her pain at the injection site using an 11-point verbal numerical rating score (VNRS) when 0 is not pain and 10 is the worst pain imaginable. None of them was heavily anesthetized and unable to give the VNRS. The residual dose of propofol was then given, followed by opioids and neuromuscular blocking agent as per usual.

In the operating room and postanesthesia care unit, each patient was carefully evaluated for paracetamol’s side effects including rash, swelling, flushing, hypotension and tachycardia.

Statistical analysis was performed by using R software 2.14.1. Continuous variables were analyzed by ANOVA F- test or Kruskal-Wallis test. Categorical variables were analyzed by ANOVA F-test, Fisher’s exact test or Chi-square test. Post-hoc analysis was carried out by using a Bonferroni correction. *P* value less than 0.05 was considered as statistical significant. Continuous variables were presented as median and interquartile range (IQR) or mean and standard deviation (SD). Categorical variables were presented as number of patients and percentages. The power of this study was 0.9.

For sample size calculation, we collected pain intensity by using the 11-point verbal numerical rating score (VNRS) in 30 patients who received propofol for an induction, without having paracetamol for premedication. The mean VNRS in this group of patients was 5.7. Anticipating that patients premedicated with paracetamol would have 25% less pain (VNRS of 4.2), a number of patients per each group was calculated to be 96. With 10% drop out, the definite number of patients per each group was 108.

## Results

**Fig. 1:**
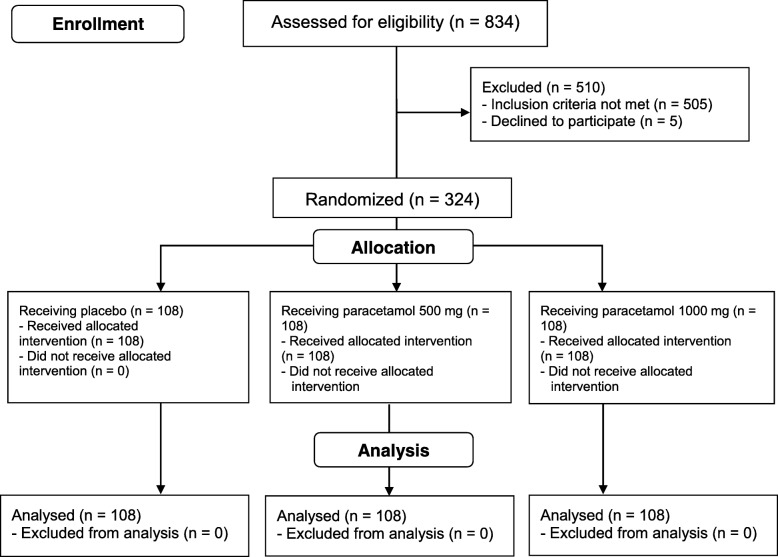
Consort flow diagram of this study

A total of 834 patients were assessed for eligibility from June 2015 to February 2016. Five hundred and ten patients were excluded and 324 patients were randomly allocated to each group. Each group equally had 108 patients. All participants were completely analyzed. (Fig. 1) There were no differences between the groups regarding gender, age, weight, height, body mass index (BMI), ASA physical classification and interval between ingestion of paracetamol and injection of propofol. (Table 1).

**Table 1:** Patient demographic data. All data are n (%) or mean (SD)

Patients	Pb (*n* = 108)	P500 (*n* = 108)	P1000 (*n* = 108)	*P*-value
Gender, n (%)				
- Male	24 (22.2)	33 (30.5)	36 (33.3)	0.16
Age (yr), mean (SD)	42.7 (11.5)	43 (12.2)	44.3 (10.3)	0.54
Weight (kg), mean (SD)	62.5 (9.6)	62.8 (9.8)	62.8 (9.1)	0.96
Height (cm), mean (SD)	159.5 (7.4)	160.3 (7.3)	159.5 (8.2)	0.67
BMI (kg/m^2^), mean (SD)	24.6 (3.6)	24.4 (3.4)	24.8 (3.5)	0.80
ASA classification, n (%)				0.19
- I	19 (17.6)	28 (25.9)	23 (21.3)	
- II	84 (77.8)	79 (73.1)	81 (75)	
- III	5 (4.6)	1 (1)	4 (3.7)	
IPP^a^, mean (SD)	65.1 (32.1)	66.5 (28.3)	70.8 (28.9)	0.35

^a^IPP = interval between ingestion of paracetamol to injection of propofol (minutes)

Data are presented as the number of patients (%) and mean ± SD values

**Fig. 2:**
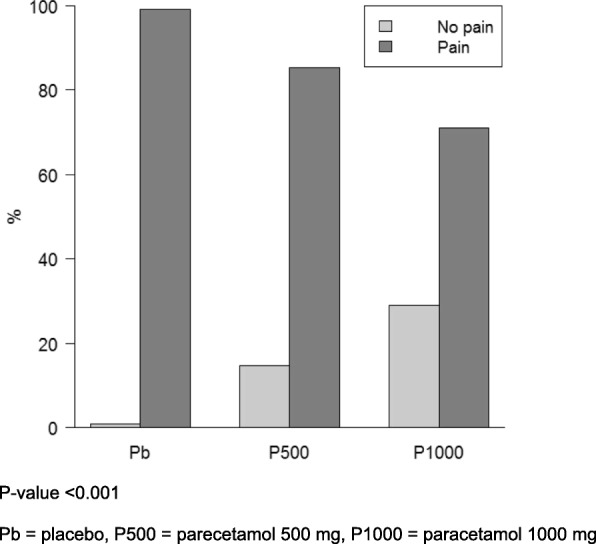
Incidence of injection pain among the 3 groups

In all cases, it was possible to achieve a clear response from the patients before they became anesthetized. The overall incidence of pain during propofol injection among the 3 groups is shown in Fig. 2. The incidence of pain was less in the P1000 group (70.4%) compared with the P500 (86.1%) and the Pb groups (99.1%) (*P* < 0.001). The incidences of pain by categories of intensity (mild/moderate/severe) were lower in the P1000 group in comparison to those in the P500 and the Pb groups (*P* < 0.001). (Table 2).

**Table 2:** Number of patients experiencing propofol injection pain among the 3 groups

Severity of pain n (%)	Pb group (*n* = 108)	P 500 group (*n* = 108)	P 1000 group (*n* = 108)
None (VNRS 0)	1 (0.9)	15 (13.9)	32 (29.6)
Mild (VNRS 1–2)	0 (0)	31 (28.7)	51 (47.2)
Moderate (VNRS 4–6)	24 (22.2)	54 (50)	25 (23.2)
Severe (VNRS 7–10)	83 (76.9)	8 (7.4)	0 (0)

*P*-value < 0.001 among the 3 groups and *P*-value < 0.001 for Pb vs P500, *P*-value < 0.001 for Pb vs P1000, *P*-value < 0.001 for P500 vs P1000

*Pb* placebo, *P500* parecetamol 500 mg, *P1000* paracetamol 1000 mg

*VNRS* verbal numerical rating score

Data are presented as the number of patients (%)

**Fig. 3:**
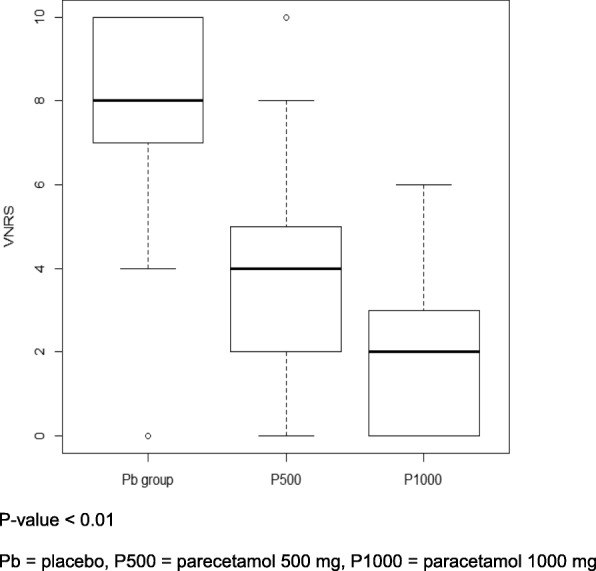
Median pain score with premedication

The median pain score showed a significant reduction in the P1000 group compared with the P500, and the Pb groups. Those were 2 (0–3), 4 (2–5), and 8 (7–10), respectively (*P* < 0.001). (Fig. 3).

There was no incidence of complications such as; rashes or edema of the tissue in each group at the recovery room.

## Discussion

In this study, we found that an oral paracetamol was effective in decreasing the incidence and severity of propofol injection pain when compared with a placebo. Premedication with 1000 mg of paracetamol was also more effective in reducing propofol injection pain than 500 mg.

Paracetamol is one of the most popular and frequently used pain killer throughout the world. The mechanisms of action are sophisticated and cover both peripheral and central antinocciceptive manners. The pain relief effect provided by paracetamol is via inhibition of the cyclooxygenase pathway centrally and peripherally, reducing the production of prostaglandins [18]. Nevertheless, its antiinflammatory effects are weak, probably due to poor effectiveness when the concentration of peroxidases is high at the area of inflammation [19]. Paracetamol has been postulated to be classified to the group of the so-called atypical NSAIDs, determined as peroxide sensitive analgesic and antipyretic drugs (PSAAD) [20]. It has been shown that paracetamol is a selective cyclooxygenase-2 inhibitor in vivo [21]. Other proposed possible modes of action are an endogenous cannabinoid effect [22, 23], fatty acid amide hydrolase (FAAH)-dependent metabolism of acetaminophen into N*-*arachidonoylphenolamine (AM404) [23], and a modulatory effect on the descending serotoninergic bulbospinal inhibitory pathway [24, 25] as concurrent administration of granisetron or tropisetron with paracetamol completely blocks the analgesic effect of paracetamol [26]. Pain relieving effect of paracetamol might also be a result of inhibition of nitric oxide (NO) formation. The synthesis of NO is through activation of L-arginine/NO pathway by substance P (SP) and N-methyl-D-aspartate (NMDA) receptors. NO is an important neurotransmitter involved in nociceptive process of the spinal cord [27, 28]. Ohashi N, et al. suggest that paracetamol metabolite N-acylphenolamine induces analgesic effect directly via transient receptor potential vanilloid 1 (TRPV1) receptors expressed on central terminals of C-fibers in the spinal dorsal horn and leads to conduction block, shunt currents, and desensitization of these fibers [29]. Treatment with paracetamol within 24 h of intensive care unit admission may lessen oxidative injury and improve renal function in adult patients with severe sepsis and detectable plasma cell-free hemoglobin [30].

Paracetamol has been found as effective for reducing propofol injection pain. Canbay et al. [15] conducted a double-blinded RCT in 150 patients and showed that the incidence of propofol injection pain was 64% in the control group and 22% in the intravenous paracetamol pretreatment group. Khouadja et al. [31] performed a double-blinded RCT in 180 patients and also showed similar results, 85% in the control group and 36.6% in the intravenous paracetamol group. Our results revealed that premedication with oral paracetamol reduced the incidence and severity of propofol injection pain. It added more information that oral paracetamol was also effective for this type of pain.

In our study, the overall incidence of pain during propofol injection was higher than other studies. The previous 3 studies [15, 31, 32] used intravenous paracetamol, not oral tablet as we did. Oral form of paracetamol exerts different pharmacokinetics and pharmacodynamics in comparison to intravenous form. This is why our results (using oral paracetamol) somewhat differed from other previous studies (using intravenous paracetamol) in terms of incidence and severity of propofol injection pain. Singla et al. [33] has shown that intravenous paracetamol has earlier and higher plasma level compared with oral paracetamol. After administration, plasma concentration of intravenous paracetamol reaches its peak rapidly within 15 min as shown by a very steep part of its graph. Plasma concentration of oral paracetamol at any time of measurement (0.25, 0.5, 0.75, 1, 2, 3, 4 and 6 h) is much lower than that of intravenous paracetamol at 15 min. The intravenous route provides a 76% higher maximum concentration (C_max_) than the oral route.

The other reasons that the incidence of propofol injection pain in the paracetamol group in previous studies was lower than ours may be from inserting a bigger venous catheter [31] and/or using a venous occlusion technique [15, 31]. It has been demonstrated that this tourniquet technique can help to increase the effectiveness of intravenous paracetamol in reducing propofol injection pain [32]. Canbay et al. [15] occluded their patient’s vein and gave pretreatment of intravenous paracetamol over 10 s. The patient’s vein was further occluded for 2 more minutes before releasing. Propofol was given after the patient’s vein had been released. Pain was measured during 5 s of paracetamol injection. The patients in Canbay et al.’s study rated their pain within the period of the highest plasma concentration of paracetamol. Regarding analgesic effects provided by intravenous in comparison with oral paracetamol, Fenlon S. et al. performed a study in 128 patients scheduled for lower third molar extraction. It has been shown that oral paracetamol is not inferior than intravenous paracetamol for providing postoperative analgesia in patients undergoing dental surgery [34].

According to the severity of pain, the incidence of mild, moderate, and severe pain was also significantly different in our P1000, P500 and Pb groups. These findings indicate that premedication with oral paracetamol reduce propofol injection pain by means of a dose-dependent fashion.

Different method of assessing pain severity may also explain our different results on severity of propofol injection pain. We used a Verbal Numerical Rating Score (VNRS) ranging from 0 to 10 (11 points) to measure our patients’ pain. All of our patients verbally reported their pain by themselves (‘subjective’ assessment). The other studies used a 4-point scale (0 = none, 1 = mild, 2 = moderate and 3 = severe). They did not mainly ask their patients to verbally rate the level of pain upon propofol injection but they principally observed their patients’ pain behaviors (‘observational’ assessment): 0 = none (negative response to questioning), 1 = mild pain (pain reported only in response to questioning with no behavioral signs), 2 = moderate pain (pain reported in response to questioning and accompanied by a behavioral sign or pain reported spontaneously without questioning), and 3 = severe pain (strong vocal response or response accompanied by facial grimacing, arm withdrawals or tears). Measuring pain by the VNRS is reliable, valid, sensitive to change, and easy to administer [35]. We did not use behavioral assessment because it is not subjective and less reliable.

This study found no adverse consequence of paracetamol.

Strengths of this study are utilization of a simple analgesic (paracetamol) and administered it to the patients in a simple way (oral route). Considering that oral paracetamol has been shown to increase the incidence of no pain as well as to reduce the incidence of severe pain upon propofol injection, the results of this study are clinically useful and applicable to daily practice because oral paracetamol is readily and widely available, practically simple and convenient to use as well as economic wise. As our study’s protocol is easy to apply and early administration or oral paracetamol is pharmacologically sensible, the result of this study can be clinically applied in general.

Limitations of this study are a subjective method of pain intensity measurement and the fractional dose of given propofol. The intensity of propofol injection pain was rated by using the verbal numerical rating score (VNRS), although patient’s self-assessment is the gold standard of pain intensity measurement but it is subjective and depends of each individual. Because propofol is a powerful induction agent, we could not inject the entire dose of propofol to each patient before measuring the pain intensity as a significant number of them felt asleep and were unable to give the pain rating.

## Conclusions

Premedication with oral paracetamol can reduce propofol injection pain on a dose-dependent basis, without causing any adverse effect. As propofol injection pain is common and remains a concern of anesthesia providers for the comfort of their patients, and early oral administration of paracetamol is pharmacologically sensible, easy to apply, well-tolerated, available and economic, the results of this study provide the basis for changing practice with a positive impact on patient care.

## Abbreviations

ALT: Alanine transminase
ASA: American Society of Anesthesiologists
AST: Aspartate transaminase
BMI: Body mass index
C_max_: Maximum concentration
COX: Cyclooxygenase
IQR: Interquartile range
NMDA: N-methyl-D-aspartate
NO: Nitric oxide
NSAIDs: Non-steroidal anti-inflammatory drugs
PSAAD: Peroxide sensitive analgesic and antipyretic drugs
SD: Standard deviation
SP: Substance P
VNRS: Verbal numerical rating score
